# Odour Maps in the Brain of Butterflies with Divergent Host-Plant Preferences

**DOI:** 10.1371/journal.pone.0024025

**Published:** 2011-08-25

**Authors:** Mikael A. Carlsson, Sonja Bisch-Knaden, Alexander Schäpers, Raimondas Mozuraitis, Bill S. Hansson, Niklas Janz

**Affiliations:** 1 Department of Zoology, Stockholm University, Stockholm, Sweden; 2 Department of Evolutionary Neuroethology, Max Planck Institute for Chemical Ecology, Jena, Germany; 3 Department of Chemistry, Royal Institute of Technology, Stockholm, Sweden; 4 Laboratory of Chemical and Behavioural Ecology, Institute of Ecology, Nature Research Centre, Vilnius, Lithuania; AgroParisTech, France

## Abstract

Butterflies are believed to use mainly visual cues when searching for food and oviposition sites despite that their olfactory system is morphologically similar to their nocturnal relatives, the moths. The olfactory ability in butterflies has, however, not been thoroughly investigated. Therefore, we performed the first study of odour representation in the primary olfactory centre, the antennal lobes, of butterflies. Host plant range is highly variable within the butterfly family Nymphalidae, with extreme specialists and wide generalists found even among closely related species. Here we measured odour evoked Ca^2+^ activity in the antennal lobes of two nymphalid species with diverging host plant preferences, the specialist *Aglais urticae* and the generalist *Polygonia c-album*. The butterflies responded with stimulus-specific combinations of activated glomeruli to single plant-related compounds and to extracts of host and non-host plants. In general, responses were similar between the species. However, the specialist *A. urticae* responded more specifically to its preferred host plant, stinging nettle, than *P. c-album*. In addition, we found a species-specific difference both in correlation between responses to two common green leaf volatiles and the sensitivity to these compounds. Our results indicate that these butterflies have the ability to detect and to discriminate between different plant-related odorants.

## Introduction

Larvae of phytophagous insects are dependent on the maternal choice of oviposition site for survival and growth. Mated females are guided to a potential host-plant primarily by visual or olfactory cues. The final close-contact evaluation of an oviposition site may rely on olfactory, gustatory or mechanical signals or a combination of those [1]. Diurnal insects as butterflies are generally believed to rely mainly on vision, while e.g. moths use olfaction to find a suitable oviposition site [2]. However, whereas the olfactory system is well studied in moths, their diurnal relatives have attracted less attention. A handful of studies have reported odorant evoked EAG responses from butterfly antennae [3], [4], [5]. However, representation of odour information in the primary olfactory centre, the antennal lobe (AL) has not previously been investigated in butterflies.

Olfactory sensory neurons (OSNs) are housed in sensillae located mainly on the antennae and labial palps [6]. All OSNs expressing a specific type of olfactory receptor (OR) converge in a spheroidal structure, glomerulus, in the AL [7], [8], [9], [10], [11]. The number of glomeruli is therefore generally equivalent to the number of different ORs. Functional imaging studies in the insect AL have shown that different odorants evoke unique patterns of activated glomeruli, which represent the activated OR types [12], [13], [14], [15], [16]. The across-neuron principle and the convergence of OSNs with identical OR type in the glomeruli create a representation of the odour world in the brain, a “spatial code”. Results from studies in both vertebrates and insects suggest common odour coding principles. Recently, it was shown that there is indeed a high similarity of coding of aliphatic compounds in ants, honeybees and rats [17]. This similarity does, of course, not exclude that species-specific adaptations have evolved to match requirements of the animal.

The butterfly family Nymphalidae contains about 6000 species with divergent host plant range [18]. We have focused on two species that differ in their degree of specialisation. The Comma butterfly, *Polygonia c-album*, is a wide generalist feeding on herbs, bushes and trees from families in four plant orders including the stinging nettle, *Urtica dioica* [19], whereas the monophagous Small Tortoiseshell, *Aglais urticae*, feeds mainly on *U. dioica* [20], [21], [22]. Specialisation on *Urticales* species seems to be an ancestral trait and a widening of host plant range took place in a lineage in which *P. c-album* belongs 20–30 million years ago [23]. This evolutionary change may have been possible due to a pre-adaptation to a polyphagous life-style or there may have been selection pressures on modification of search mechanisms, e.g. olfaction, after a host shift. From earlier studies we know that *P. c-album* has a reduced ability to discriminate between different qualities of *U. dioica* compared to two specialist species, *Inachis io* and *Vanessa indica* [24]. In addition, an English population of *P. c-album* is more specialised than the Swedish population and is actually better at discriminating *U. dioica* from a non-host plant, *Lamium album* [25], [26]. In these behavioural studies it was not investigated if discrimination was based on olfactory or other sensory cues.

The aim of the present study was to investigate the representation of olfactory information in the ALs of the two butterfly species by means of Ca^2+^ imaging. The major questions we wanted to address were if the butterflies responded to plant related volatiles in an odour-specific manner, i.e. with specific combinations of activated glomeruli and if responses differed between the two species.

## Results

### Antennal lobe morphology is similar in the two species

**Figure 1:**
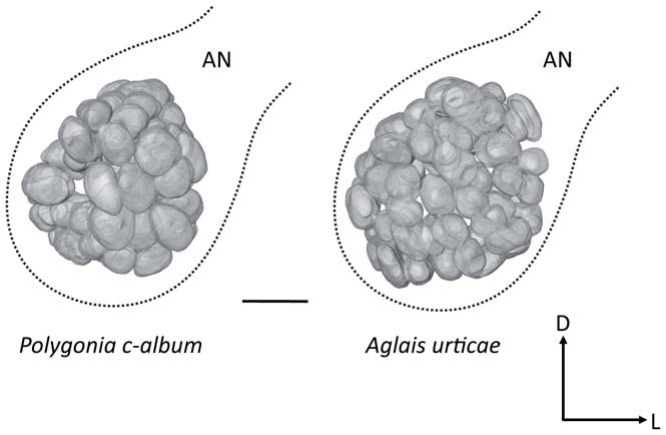
Antennal lobe reconstructions. Frontal views of surface reconstructions based on optical sections of the ALs. Outlines show the borders of the ALs. L, lateral; D, dorsal. Scale bar  = 50 µm. AN, antennal nerve.

First we performed an anatomical investigation of the ALs in the two species. Figure 1 shows surface reconstructions of the ALs after immunostaining with anti-synapsin. Both species have 60–65 glomeruli (n = 5 of each species). The glomerular diameter (widest) was first measured in all visible glomeruli and then averaged in each animal. The median diameter across animals was 46.5±2.2 µm (n = 5 animals) in *P. c-album* and 48.2±1.6 µm in *A. urticae* (n = 5). Glomerular diameter did not differ between the species (p = 1.0; Mann-Whitney-U test). Thus, the morphology of the ALs in the two species is very similar and makes these species appropriate for a physiological comparison.

### Responses to plant extracts are more specific in *A. urticae*

**Figure 2:**
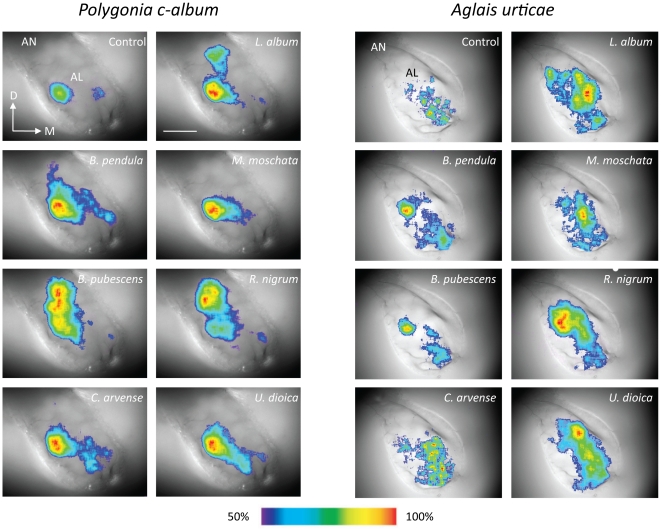
Responses to plant extracts. False-colour coded images of odour-evoked activity to plant extracts and control (solvent) superimposed on wide-field images of the lobes. Each response is scaled to the upper 50% of its intensity range. D, dorsal; M, medial. AN, antennal nerve; AL, antennal lobe. Scale bar  = 100 µm.

Secondly, we used Ca^2+^ imaging to study AL responses to antennal stimulation with plant extracts and single compounds. Figure 2 shows typical examples from one individual of each species of false-colour coded images of responses to the plant extracts superimposed on wide-field images of the lobes. Each response is scaled to its own intensity range and the false-colour code ranges from 50% of activity to maximal activity. All extracts activated multiple glomeruli. The solvent cyclohexane also evoked responses but much weaker than the plant extracts.

**Figure 3:**
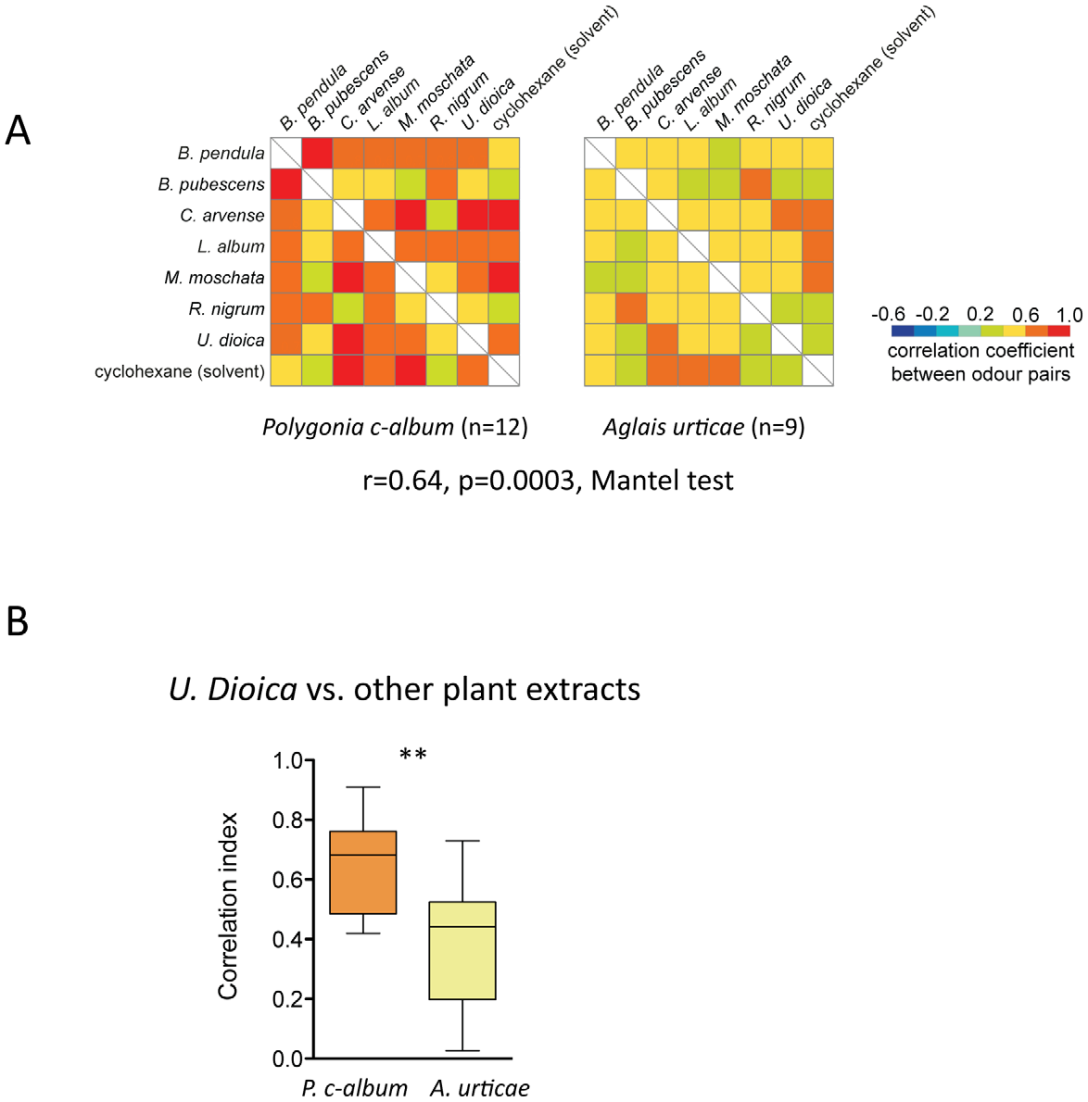
Responses to plant extracts. A Similarity matrices of responses to plant extracts in *P. c-album* (left, n = 12) and *A. urticae* (right, n = 9). The colour coding depicts the median correlation index according to the scale. **B** The graph shows the correlation between responses to *U. dioica* and responses to all other plant extracts in the two species (** p<0.001; Mann-Whitney-U test). A box plot shows the median correlation index of a pair of odorants (horizontal line), the 25th and 75th percentile (lower and upper margin of the box) together with the minimum and maximum values (whiskers). Colour coding as in A.

We calculated correlation indices between all pairs of responses to plant extracts. The similarity matrices in Figure 3A show the median correlation values for *P. c-album* and for *A. urticae*. The matrices were significantly correlated with each other (r = 0.64, p = 0.0003, Mantel test). However, visual inspection of the similarity matrices show that the responses were often less similar in *A. urticae* than in *P. c-album.* Next we calculated the correlation between responses to *U. dioica* and responses to all other plant extracts. The correlation coefficients were significantly lower in *A. urticae* than in *P. c-album* (Fig. 3B; p<0.001; Mann-Whitney-U test).

### Responses to single compounds are generally similar between the species

**Figure 4:**
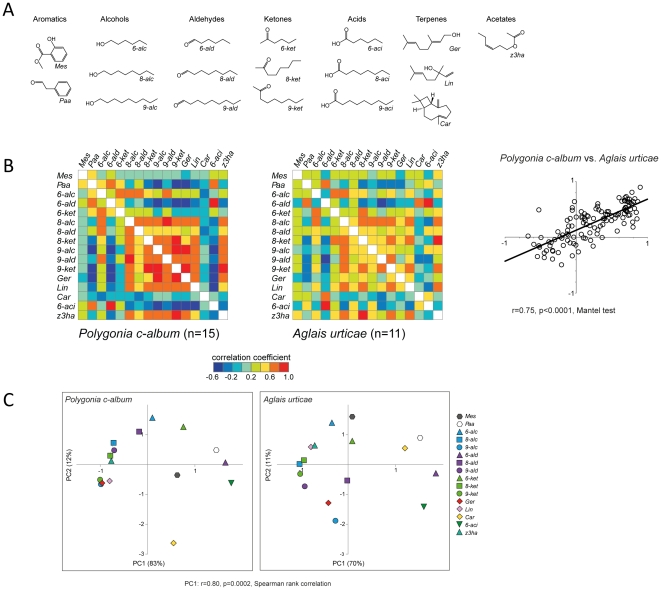
Responses to single compounds. **A** Molecular structures of the tested plant related compounds. **B** Similarity matrices of responses to these compounds. Median correlation values are colour-coded according to scale. Correlation between the species r = 0.75 (p<0.0001, Mantel test). **C** A principal component analysis showing the first two components for each species. Correlation of the first component (PC1) r = 0.80 (p = 0.0002, Spearman rank correlation).

We used 18 different plant-related compounds (Fig. 4A). All odorants except octanoic acid and nonanoic acid elicited reliable and repeatable responses in all animals. Therefore, we excluded these two compounds in the following analyses. The remaining 16 odorants evoked activity in different combinations of glomeruli. Based on pairwise correlations of all odorants we constructed similarity matrices (Fig. 4B). These matrices were significantly correlated with each other (r = 0.75, p<0.0001, Mantel test). In addition, we did a principal component analysis. Figure 4C show graphic representations of the first two principal components, which describe 94% and 81% of the total variance of the data, respectively. The first component is significantly correlated between the species (r = 0.80; p = 0.0002; Spearman rank correlation). That is, in general odour-evoked activity is highly similar in the two species.

### Relation between structural similarity and neural response similarity

**Figure 5:**
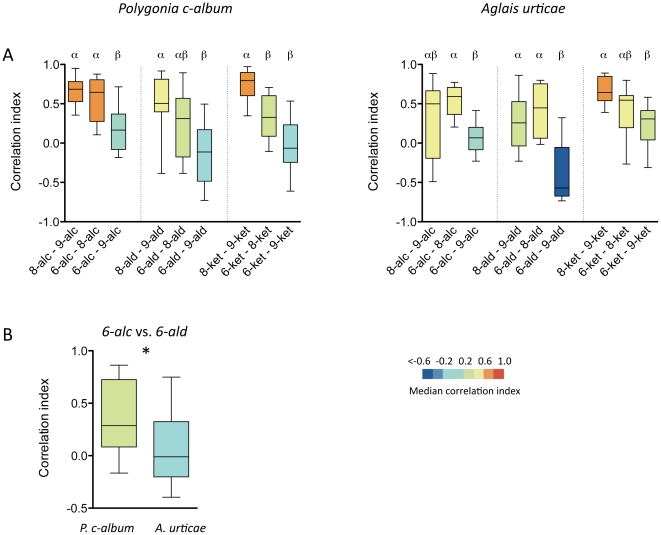
Correlation between chemical structure and response. **A** Pairwise correlations between compounds with the same functional group but different chain length. In each group (alcohols, aldehydes or ketones) in a graph the chain length of the compounds differ by 1, 2 or 3 carbon atoms, respectively. Bars capped by different letters differ significantly within the chemical group (Kruskal Wallis test followed by Dunn's Multiple Comparison Test). A box plot shows the median correlation index of a pair of odorants (horizontal line), the 25th and 75th percentile (lower and upper margin of the box) together with the minimum and maximum values (whiskers). The colour coding depicts the median correlation index according to the scale. *A. urticae* (n = 10) and *P. c-album* (n = 15). **B** Correlation of responses to two compounds (6-alc and 6-ald) with the same chain length but different functional group (* p<0.05; Mann-Whitney-U test).

To assess the relation between molecular structure and neural response we used an odour panel consisting of three series of aliphatic compounds (alcohols, aldehydes and ketones) with differing chain lengths (6, 8 or 9 carbon atoms). We calculated pairwise correlations between compounds with the same functional group but different chain length. With the exception of alcohols in *A. urticae*, responses to two compounds differing by only one carbon atom were more correlated than compounds differing by 3 carbon atoms, disregarding the functional group (Fig. 5A; Kruskal Wallis test followed by Dunn's Multiple Comparison Test). In *A. urticae*, however, responses to alcohols differing by 2 carbon atoms differed from responses to alcohols differing by 3 carbon atoms. That is, compounds with similar chemical structure generally evoked more similar activity patterns than compounds with less similar structures.

In addition, we compared responses to compounds sharing chain length but with different functional groups. The correlation between responses to *6-alc* and *6-ald* was significantly lower in *A. urticae* than in *P. c-album* (Fig. 5B; p<0.05; Mann-Whitney-U test). No other pairs were significantly different.

### Species differences in sensitivity to green leaf volatiles

**Figure 6:**
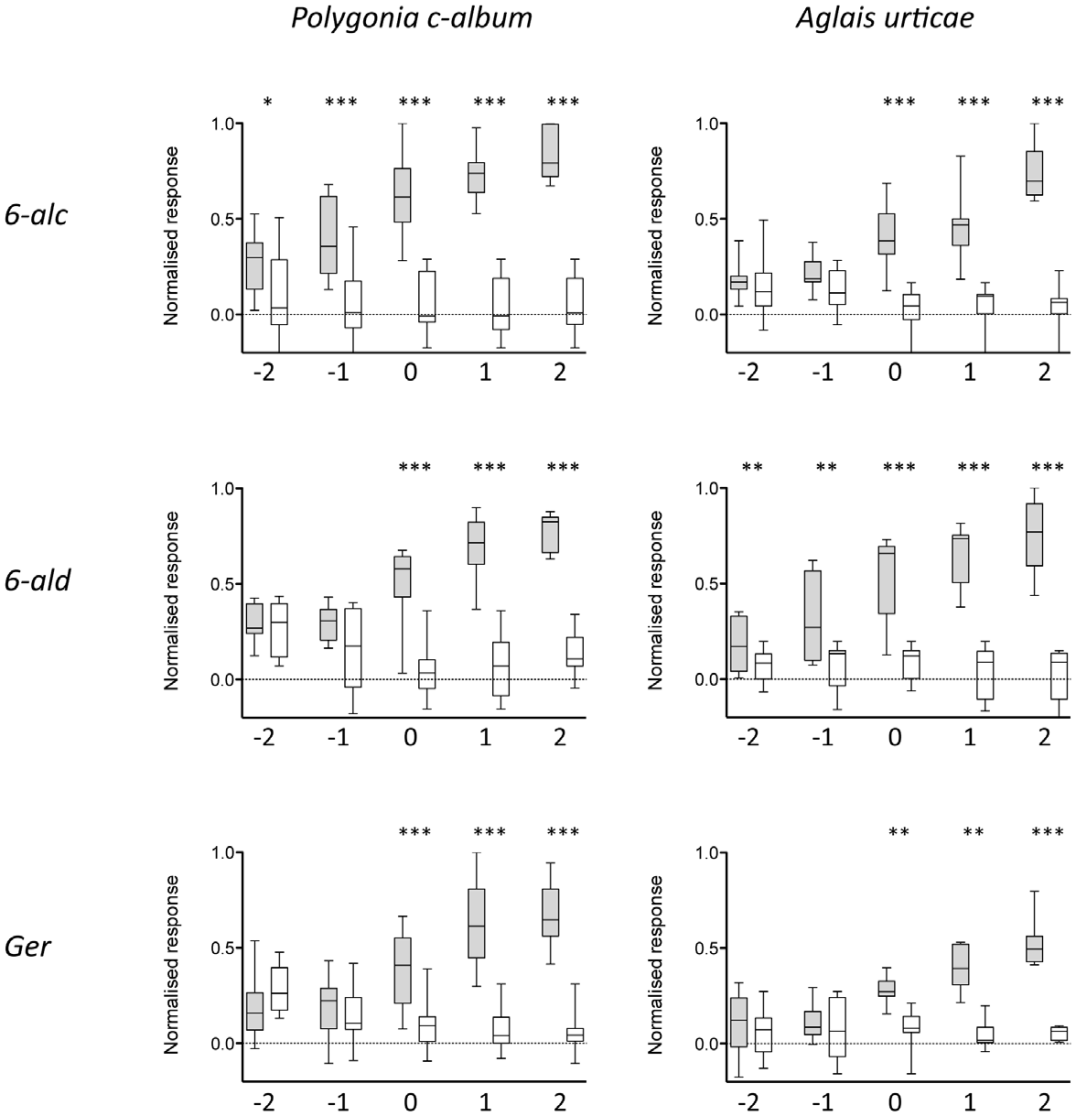
Dose-response correlations. Responses to hexan-1-ol, hexan-1-al and to geraniol at different doses in *P. c-album* (n = 14, 8 and 9, respectively) and *A. urticae* (n = 11, 8 and 7, respectively). The x-axes depict the doses; −2–2 means 4.8×10^−2^–4.8×10^2^ µg (*6-alc* and *6-ald*) or 5.2×10^−2^–5.2×10^2^ µg (*Ger*). Response values are normalised to the strongest response in each animal. The glomerulus with the strongest activity at each dose is compared with the response to the solvent in that particular glomerulus. A response to an odorant (grey bar) significantly differing from a response to the solvent (white bar) is depicted with asterisks above the bars (two-tailed Wilcoxon matched-pairs signed rank test; * p<0.05, ** p<0.01, *** p<0.001). A box plot shows the median normalised response to an odorant (horizontal line), the 25th and 75th percentile (lower and upper margin of the box) together with the minimum and maximum values (whiskers).

In the test above we found that responses to two commonly occurring green leaf volatiles, GLVs [1], *6-alc* and *6-ald* differed between the species. Therefore we tested sensitivity over a range of doses to these compounds and, as a control, to the floral compound geraniol (Fig. 6). We identified the glomerulus with the strongest activity at each dose and compared this response to the response to the solvent (two-tailed Wilcoxon matched-pairs signed rank test). *P. c-album* responded to *6-alc* down to 4.8×10^−2^ µg compared to 4.8×10^0^ µg for *A. urticae.* With *6-ald* the sensitivity was reversed with *A. urticae* being more sensitive than *P. c-album* (4.8×10^−2^ µg and 4.8×10^0^ µg, respectively). Sensitivity to geraniol was similar in the two species (threshold 5.2×10^0^ µg).

In summary, we found that responses to plant extracts and single plant related compounds were generally similar in the two species. However, we showed that *A. urticae* responded more discriminatively than *P. c-album* to the common host plant *U. dioica.* In both species we found a correlation between structural similarity and the similarity of responses, whereas both correlation between hexan-1-ol and hexan-1-al and sensitivity to these compounds differed between the species.

## Discussion

Searching for a suitable host plant may involve input from different sensory modalities. As opposed to e.g. moths, diurnal insects as butterflies are believed to mainly rely on vision when trying to locate an oviposition site [2]. However, in the present study we showed that two species of Nymphalid butterflies have the neuronal capacity to respond to extracts of host- and non-host plants and to a number of plant-derived compounds. This is the first study showing odour-evoked activity in the butterfly AL. Not surprisingly, we found that the tested compounds generally evoked unique combinations of activated glomeruli. Furthermore, even though responses to the tested odorants generally were similar between the species we also found species-specific differences.

Despite that butterflies diverged from moths at least 100 Ma ago [27], [28] and that they have adapted to a diurnal lifestyle, anatomical and physiological characteristics of the olfactory system seem to be conserved. The ALs of the butterflies in our study consist of 60–65 glomeruli, which is numerically comparable to moths [29], [30], [31], [32]. To our knowledge, the only previously published anatomical study of the AL of butterflies showed that the number of glomeruli in *Pieris brassicae* is 62±3 [33]. If we can extrapolate the rough one-to-one relationship of receptors to glomeruli in *Drosophila* [8], [11] we can assume that these butterflies have approximately 60 receptor types. The odour-evoked responses in the AL showed similarities to what has been observed in other animals [13], [14], [15], [16], [17], [34], [35]. Most of the odorants evoked unique, though overlapping, patterns of activated glomeruli. Another feature that has been observed in several species is the correspondence between structural similarity and the similarity of neural responses [17], [36], [37]. One aspect of structural feature that can be varied systematically is chain length. We showed that the distance between compounds with differing chain length generally corresponded with the similarity of glomerular responses.

Even though the similarity matrices in Figure 2 did not differ significantly between the two butterfly species we found that responses to stinging nettle, *U. dioica*, was less similar to responses to other plant extracts in *A. urticae* than in *P. c-album*. Whether this neural response similarity also corresponds to perceptual similarity [38], [39], [40] remains to be investigated. Even though *A. urticae* is more discriminative when choosing a host plant than *P. c-album* [20], [21], [22] we do not know if the choice is based on olfaction or other sensory input. In a recent study it was, however, demonstrated that another butterfly species, *P. rapae*, could behaviourally distinguish a host plant from a non-host plant exclusively by olfactory (but not visual) cues [41].

Both species responded to all single compounds except octanoic and nonanoic acid. The reason for the lack of measurable responses to these compounds may be either that the butterflies do not possess receptors that can be activated, at least not at the concentrations used in this study. An alternative explanation is that glomeruli innervated by OSNs responding to these acids are located on the caudal side of the AL, which is not accessible for imaging. Responses to single compounds were generally similar between the species. We found, however, that the correlation between responses to two common GLVs differed between the species in that glomerular activity patterns evoked by these compounds were less similar in *A. urticae* than in *P. c-album*. We further tested if sensitivity to these GLVs differed between the species and found that *P. c-album* was more sensitive to a six-carbon alcohol than *A. urticae*. For a six-carbon aldehyde the situation was reversed, with *A. urticae* being more sensitive of the species. When we tested the sensitivity to a floral compound, which probably has no relevance for host plant search, there was no difference between the species. Green leaf volatiles are emitted in small amounts by intact green leaves [1] and it has been demonstrated that these compounds are important cues for many insects when trying to locate and identify a host plant. For example, in the Colorado beetle, *Leptinotarsa decemlineata*, a correct ratio between GLVs emitted from potato leaves is essential for eliciting positive anemotaxis [42]. The plants used in our study emit GLVs in different quantities and ratios. The two birch species, e.g. emit *6-ald* and *6-alc* at markedly different ratios [43]. Preliminary data show that *U. dioica* emits more *6-ald* than *6-alc* compared to *R. nigrum* and the *Betula* species ([43], R Mozuraitis, personal communication). It should be noted that it is very difficult to compare release of volatiles from different plants. Sampling methods, all kinds of stress, seasonal variations and even variations within different parts of a plant are factors that may influence the analysis of released volatiles. However, in the present study we always used the same extracts in both species and the fact remains that *A. urticae* and *P. c-album* responded differently.

The diurnal lifestyle of butterflies and the evolutionary divergence from moths more than 100 Ma ago [27], [28] may have generated some differences in the olfactory system. However, the morphology of the AL is similar and most of odorants used in this study has also been shown to elicit responses in different moth species [12], [13]. As moths are well-known for their extreme sensitivity to odorants [44] it would be interesting to compare it to butterflies. In a study investigating concentration dependency of glomerular activity patterns in a moth [45] using the same methodology as in our study it was shown that the lowest dose of the floral compound geraniol the moth responded to was 0.1 µg. In our study we observed a significant response at 4.8 µg but not at 0.48 µg. This indicates that moths may be more sensitive, at least to certain odorants.

It has been suggested that odour quality is encoded in a spatial across-glomeruli (and thus across-OSN) code in the AL, e.g. [33], and that this code can be visualised by different functional imaging techniques [15], [16]. Studies in different species have shown that similarity of neural responses is correlated with perceptual similarity of odorants [38], [39], [40]. The more similar the combinatorial activity patterns the more difficult it is to discriminate between the odorants. Hence, our results indicate that *A. urticae* may have a higher ability to perceptually distinguish odours of a host plant from non-host plants and also *6-alc* from *6-ald* than *P. c-album*. However, if glomerular activity patterns observed in butterflies can be related to behaviour needs to be examined in future studies. As bath application with a Ca^2+^-sensitive dye is believed to mainly reflect input activity in the AL [46], [47] it would be very interesting to investigate how olfactory information is processed and finally shaped in the PNs. It is likely that differences between species may be further enhanced due to shaping by the AL network. For example, the level of neuroactive substances in the AL may vary considerably, even during development of an individual [48], [49]. Therefore, we may assume that larger differences between species may have evolved concerning release and signalling with transmitters and modulators in the AL than the responsiveness of the ORs.

In conclusion, odour-evoked activity in the ALs of two species of nymphalid butterflies with diverging host plant range was combinatorial across glomeruli and mostly odour unique. This means that the butterflies not only have the ability to detect but it also indicates that they may be able to discriminate between plant-related odorants. Even though responses were often similar across the species, the more specialised of the tested species, *A. urticae*, responded more discriminatively to certain host plant-related odours than *P. c-album.* Future studies will hopefully reveal if our findings have a behavioural relevance. We have, however, demonstrated that the butterflies have a neural substrate for detection and discrimination of plant-related volatiles and that there are species-specific differences that may be evolutionarily important.

## Materials and Methods

### Animals

Two species of butterflies belonging to the family Nymphalidae were used in the experiments. These were the Comma Butterfly, *Polygonia c-album* (Lepidoptera: Nymphalidae; Linneus), and the Small Tortoise Shell, *Aglais urticae* (Lepidoptera: Nymphalidae; Linneus). The parental generation of *P. c-album* and *A. urticae* were caught in the vicinity of Stockholm, Sweden and their offspring was used. No permits were required in accordance with the Swedish principle of public access to the wilderness. Both mated and virgin females were used of *P. c-album* whereas only virgin females were used of *A. urticae.* All animals were between 3–5 weeks old when tested and the mated females were tested at least 2 weeks after mating. We did not detect any difference in response between mated and virgin *P. c-album* when we tested them separately, which justified pooling of data in the subsequent analyses. All animals were kept separated in a low temperature (9°C) chamber (12/12-h light/dark cycle) until start of experiment and were fed with sugar water *ad libitum*. Animals were moved to room temperature 24 hrs prior to the start of the experiment.

### Immunostaining and AL reconstruction

Brains of butterflies were dissected in phosphate-buffered saline with 0.25% Triton-X (PBS-Tx) and fixed overnight at 4°C in 4% paraformaldehyde in 0.1 M sodium phosphate buffer. After careful rinsing in PBS-Tx the brains were preincubated overnight in 5% normal goat serum in PBS-Tx. Brain tissue was incubated for 48-72 hours in mouse monoclonal anti-synapsin (anti-SYNORF1, 1∶10; Developmental Studies Hybridoma Bank, Iowa City, IA.). For detection of antiserum we used a Cy3-tagged secondary antibody (1∶100; Jackson ImmunoResearch, West Grove, PA).

Brains were dehydrated in a series of increasing ethanol concentration (50–100%) and finally cleared and mounted in methyl salicylate. The preparation was scanned with a Zeiss LSM 510 META (Zeiss, Jena, Germany) confocal laser scanning microscope and images were obtained at optical section thickness of 0.27–0.31 µm with a 40x oil objective. Three-dimensional surface reconstructions of confocal stacks were generated using the segmentation tool in AMIRA 4.1 software (Mercury Computer Systems GmbH, Berlin, Germany) and edited for contrast and brightness in Adobe Photoshop CS4 Extended (v. 11.0, San Jose, CA).

### Preparation and staining

Animal preparation was similar to previous studies in moths [13], [45], [50]. Briefly, animals were placed in a 1000 µl pipette tip with the tip cut open to fit the head. The protruding head at the narrow end was fixed in this position with dental wax. Labial palps and proboscis were removed to reduce movements during the experiments. A window was cut in the head capsule between the compound eyes and the tissue covering the brain was removed to uncover the antennal lobes. The membrane-permeant fluorescent calcium indicator (Calcium Green-2 AM, Molecular Probes) was dissolved in physiological saline [51] with 20% Pluronic F-127 (Molecular Probes) to a final concentration of 30 µM. A drop of this dye solution was bath applied to the exposed brain and the preparation was incubated for about 60 min at 4°C. The brain was subsequently rinsed several times with physiological saline to remove excessive dye. Bath application with Calcium Green-2 AM potentially stains different types of cells in the AL. However, odour-evoked responses are supposed to originate mainly from OSNs [46], [47].

### Optical imaging

The imaging set-up consisted of an air-cooled 12-bit slow-scan CCD camera (Olympus U-CMAD3) mounted to an upright microscope (Olympus BX51WI) equipped with a water immersion objective (Olympus, 20x/0.95). Calcium green-2 AM was excited at 475 nm (500 nm SP; xenon arc lamp, Polychrome V, Till Photonics) and fluorescence was detected at 490/515 nm (DCLP/LP). The set-up was controlled by the software Tillvision 4.0 (Till Photonics). Four-fold symmetrical binning resulted in image sizes of 344×260 pixels with one pixel corresponding to an area of 1.25 µm×1.25 µm.

### Odour stimuli

We used a setup of 18 odorants (Sigma-Aldrich) belonging to different structural groups. These were: a) aromatics: methyl salicylate (*Mes*), CAS-#: 119-36-8, phenyl acetaldehyde (*Paa*), CAS-#: 122-78-1; b) alcohols: hexan-1-ol (*6-alc*), CAS-#: 111-27-3, octan-1-ol (*8-alc*), CAS-#: 111-87-5, nonan-1-ol (*9-alc*), CAS-#: 143-08-8; c) aldehydes: hexanal (*6-ald*), CAS-#: 66-25-1, octanal (*8-ald*), CAS-#: 124-13-0, nonanal (*9-ald*), CAS-#: 124-19-6; d) ketones: 2-hexanone (*6-ket*), CAS-#: 591-78-6, 2-octanone (*8-ket*), CAS-#: 111-13-7, 2-nonanone (*9-ket*), CAS-#: 821-55-6; e) acids: hexanoic acid (*6-aci*), CAS-#: 142-62-1, octanoic acid (*8-aci*), CAS-#: 124-07-2, nonanoic acid (9-aci), CAS-#: 112-05-0; f) terpenes: geraniol (*Ger*), CAS-#: 106-24-1, (±)-linalool (*Lin*), CAS-#: 78-70-6, β-caryophyllene (*Car*), CAS-#: 87-44-5 and g) acetates: Z3-hexenyl acetate (*z3ha*), CAS-#: 3681-71-8. Purity of the compounds was >97% (except *9-alc*: 95%, *Paa*: 90%, *Ger*: 96%. All odorants were diluted in mineral oil (*Mol*, Sigma-Aldrich) to a ratio of 1∶100 (vol/vol). For the dose-response experiment odorants were diluted in decadic steps (from 1∶100000 to 1∶10). These dilations correspond to 4.8×10^−2^–4.8×10^2^ µg of *6-alc* and *6-ald* and µg 5.2×10^−2^–5.2×10^2^ µg of *Ger* when 6 µl were applied to a filter paper.

In addition, we used extracts of seven different plants: *Betula pendula* (N), *Betula pubescens* (P), *Cirsium arvense* (N*), *Lamium album* (N), *Malva moschata* (N*), *Ribes nigrum* (P) and *Urtica dioica* P, A). Abbreviations in brackets denote non-host plants (N) or natural host plants of *P. c-album* (P) and *A. urticae* (A), respectively. The plant species denoted with an asterisk are host plants to related nymphalid species [22]. Intact leaves of plants (total area 40 cm^2^) were removed from the stems and placed in 200 ml glass beakers containing ether. The leaves were handled carefully to reduce damage. The solution was left to evaporate and subsequently filtered and dissolved in cyclohexane. The same extracts were used to test responses in all animals.

### Odorant stimulation

Six µl of the diluted odorants or plant extracts were applied onto a circular piece of filter paper (diameter: 12 mm); 6 µl of either mineral oil (single compounds) or cyclohexane (extracts) served as control stimuli. Filter papers were inserted into glass Pasteur pipettes and were renewed every day. A humidified and charcoal-filtered continuous air stream (1 l/min) was ventilating the antenna ipsilateral to the recorded AL through a glass tube (5 mm inside diameter). The glass tube ended ∼10 mm from the distal part of the antenna. An empty Pasteur pipette was inserted through a small hole in the glass tube, blowing an air stream of 0.1 l/min. Another air stream (0.1 l/min) was blown through the odour-laden pipette by a computer-triggered puffer device (Syntech, Hilversum, The Netherlands) during 2 s (starting at frame 8) into the continuous stream of air. During stimulation, the air stream was switched from the empty pipette to the odour-laden one in order to minimize the influence of added air volume. One odorant stimulation experiment lasted 10 s and was recorded with a sampling rate of 4 Hz corresponding to 40 frames. The time course was as follows: 2 s clean airstream (frame 1–8), 2 s stimulus airstream (frame 9–16), and 6 s clean airstream (frame 17–40). We used at least 60 sec interstimulus periods to reduce adaptation.

### Analysis and statistics

With the software Till-Vision we constructed false-colour coded images of relative changes of fluorescence intensity during the peak time of activity. By visual inspection of activity maps elicited by all odorants we drew circular regions of interest (ROI, 20 pixels diameter corresponding to 25 µm, which is roughly 50% of the diameter of an average glomerulus) round the centres of activity. This resulted in 14–17 ROIs in all animals. An additional ROI was drawn in an area with minimal activity, which served as a control. In an earlier study in moths it was demonstrated that activity foci correspond to individual glomeruli [45]. The mean pixel value within a ROI was calculated for each time-point (40 frames) in a sequence and exported to Microsoft Excel. In Excel we first made a temporal median filtering of data over three consecutive frames. Secondly we calculated the relative fluorescence (dF/F) where F was defined as the mean value of frames 2–7. To correct for bleaching we subtracted the values of the control ROI from the values of all other ROIs for each recording. A response was finally defined as the mean of frames 12–15 (peak of activity). This means that for every odorant we have a response profile with 14–17 response values that correspond to the relative changes in fluorescence intensity in the ROIs.

Correlation values were calculated based on the response profiles (above) with JMP 8 (SAS Institute Inc.). Statistical analyses were made using Prism 5 (GraphPad Software, Inc.), XLSTAT (Addinsoft), and PAST (Hammer and Harper).
